# Attitudes of social workers and their patients concerning early identification of alcohol-related problems

**DOI:** 10.1186/1940-0640-7-S1-A40

**Published:** 2012-10-09

**Authors:** Elina Renko

**Affiliations:** 1 Department of Social Research, University of Helsinki, Helsinki, Finland

## 

This qualitative study compared social workers’ and their patients’ attitudes toward screening for alcohol-related problems. Existing studies on attitudes toward alcohol screening have largely been quantitative and have typically been conducted in primary-care settings. The aim of this qualitative research was not to make generalizations from sample to population but to analyze plurality of viewpoints with regard to screening. Social workers (n = 14) and their customers (n = 14) were interviewed. All interviewees were asked to comment on the following statement: It is stigmatizing to ask a client, who is not a heavy drinker, about their alcohol consumption. Both social workers and their patients presented a range of positive, reserved, and negative attitudes towards the statement. Participants in both groups justified their agreement with the statement by referring to the assumption that asking about alcohol consumption implies a suspicion of heavy drinking. Social workers also connected this suspicion to a stereotypical view of social work clients and thought their patients would want to avoid such stigmatization. Reserved attitudes of social workers and their patients were justified from the viewpoint that, even if they personally did not perceive asking about alcohol consumption to be stigmatizing, others might think so. Finally, while justifying their negative attitudes towards the statement, both groups considered asking about alcohol consumption to be an integral part of social work. These findings suggest that social workers and their patients have similar attitudes toward the alcohol screening. Further research is warranted.

